# A New Sturgeon Herpesvirus from Juvenile Lake Sturgeon *Acipenser fulvescens* Displaying Epithelial Skin Lesions

**DOI:** 10.3390/pathogens12091115

**Published:** 2023-08-31

**Authors:** Sharon Clouthier, Marek Tomczyk, Tamara Schroeder, Cheryl Klassen, André Dufresne, Eveline Emmenegger, Thomas Nalpathamkalam, Zhuozhi Wang, Bhooma Thiruvahindrapuram

**Affiliations:** 1 Freshwater Institute, Department of Fisheries and Oceans, 501 University Crescent, Winnipeg, MB R3T 2N6, Canada; tamara.schroeder@dfo-mpo.gc.ca; 2 Manitoba Agriculture & Resource Development Veterinary Diagnostic Services, 545 University Crescent, Winnipeg, MB R3T 5S6, Canada; marek.tomczyk@gov.mb.ca; 3 Manitoba Hydro, 360 Portage Ave, Winnipeg, MB R3C 0G8, Canada; cklassen@hydro.mb.ca; 4 National Centre for Foreign Animal Disease, Canadian Food Inspection Agency, 1015 Arlington Street, Winnipeg, MB R3E 3M4, Canada; andre.dufresne@inspection.gc.ca; 5 Western Fisheries Research Center, U.S. Geological Survey, 6505 NE 65th Street, Seattle, WA 98115, USA; eemmenegger@usgs.gov; 6 The Centre for Applied Genomics, The Hospital for Sick Children, 686 Bay Street, Toronto, ON M5G 0A4, Canada; thomas.nalpathamkalam@sickkids.ca (T.N.); zwang@sickkids.ca (Z.W.); bthiruv@sickkids.ca (B.T.)

**Keywords:** Acipenser herpesvirus, AciHV-3, *Alloherpesviridae*, epidermal hyperplasia, next-generation sequencing, NGS, Bayesian phylogenetics, genotyping, Lake Sturgeon

## Abstract

Herpesvirus infections of sturgeon pose a potential threat to sturgeon culture efforts worldwide. A new epitheliotropic herpesvirus named Acipenser herpesvirus 3 (AciHV-3) was detected in hatchery-reared Lake Sturgeon *Acipenser fulvescens* displaying skin lesions in central Canada. The growths were discovered in the fall, reached average prevalence levels of 0.2–40% and eventually regressed. No unusual mortality was observed. The cellular changes within the lesions included epithelial hyperplasia and were reminiscent of other herpesvirus infections. The virus was not evident in lesions examined by electron microscopy. Skin tissue homogenates from symptomatic sturgeon produced atypical cytopathic effects on a primary Lake Sturgeon cell line, and next-generation sequence analysis of the DNA samples revealed the presence of an alloherpesvirus. A new genotyping PCR assay targeting the major capsid protein sequence detected AciHV-3 in symptomatic Lake Sturgeon as well as other apparently healthy sturgeon species. Bayesian inference of phylogeny reconstructed with a concatenation of five alloherpesvirus core proteins revealed a new *Alloherpesviridae* lineage isomorphic with a new genus. The presence of AciHV-3 homologs in cell lines and sturgeon sequence datasets, low sequence divergence among these homologs and branching patterns within the genotyping phylogeny provide preliminary evidence of an endogenous virus lifestyle established in an ancestral sturgeon.

## 1. Introduction

Herpesviruses (HV) have been detected in a number of sturgeon species, including Lake Sturgeon *Acipenser fulvescens,* and pose a potential threat to the sustainability of commercial and conservation efforts undertaken for acipensers worldwide. Lake Sturgeon belong to an ancient lineage of the *Acipenseridae* family that diverged from Paddlefish (*Polyodontidae*) of the Acipenseriforms order between 155 and 204 million years ago [1,2,3,4]. Extant Lake Sturgeon spend their entire lives in fresh water and, in Canada, are located in the Hudson Bay and Atlantic drainage basins [5]. Across this geographic range, designatable units of this species are classified as Special Concern, Threatened, or Endangered by the Committee on the Status of Endangered Wildlife in Canada [5]. Conservation programs play a critical role in supplementing these at-risk stocks and use a variety of strategies including repatriation of hatchery-reared progeny back to the natal river of their wild-caught parents [6].

Outbreaks of herpesvirus disease in sturgeon were originally described in wild White Sturgeon *A. transmontanus* in California and involved two types of acipenser herpesviruses (AciHV)—AciHV-1 and AciHV-2 [7,8]. Since then, homologues of these viruses have been found in cultured and wild White Sturgeon in other parts of the USA and Italy [9,10], hatchery-reared Shortnose Sturgeon *A. brevirostrum* in Canada [11], farmed Siberian Sturgeon *A. baerii* and Bester (beluga *Huso huso* × sterlet *A. ruthenus* hybrid) in Russia [12,13,14] and wild Lake Sturgeon in American tributaries of the Great Lakes [15,16]. A third sturgeon herpesvirus was reported by Kelley et al. [17] and then retracted in Kurobe et al. [10]. Similar to other herpesviruses, AciHV-1 and AciHV-2 display a high degree of host species specificity, and the same host species can be infected with either virus [7,8,18]. Sturgeon herpesviruses are taxonomically classified to the *Alloherpesviridae* family of the order Herpesvirales [10,19,20]. The International Committee on Taxonomy of Viruses (ICTV) recognizes four genera under this family: *Batravirus*, *Cyvirus*, *Ictavirus* and *Salmovirus* [21]. AciHV-2 form a distinct group (i.e., *Ictavirus acipenseridallo2* species) in the *Ictavirus* genus, whereas AciHV-1 remains unclassified [10,15,16]. The genome sequence of AciHV-2 has yet to be published, but preliminary analysis of a Shortnose Sturgeon AciHV-2 isolate indicates an approximate length of 167 kb [22]. The Lake Sturgeon AciHV-1 genome is 202,660 bp and encodes the twelve core genes shared by all alloherpesviruses [16,23].

Sturgeon herpesviruses are epitheliotropic and associated with chronic, recurrent skin infections in wild and hatchery populations [24,25]. As with other herpesviruses, their recurrent biphasic lifecycle is characterized by a primary infection that can manifest as an acute disease leading to morbidity and mortality or as a mild infection in which fish remain asymptomatic or display benign skin growths referred to as lesions or tumors [7,8,9,12,14,15,26]. The second phase of the herpesvirus cycle involves establishment of a persistent infection through latency, either by circularization of the virus genome in the nuclei of (non)replicating cells or by integration of the virus genome into the host chromosome [27]. Although little is known about how sturgeon herpesviruses establish a latent infection, lesion recurrence in convalescent sturgeon after their recovery, from either an acute or mild infection, provides evidence that AciHV-2 can establish a persistent infection [8]. The risk factors that influence the (re)occurrence and severity of AciHV disease include virus strain, virus dose, fish age, tank stocking density and water flow [8,14,15,26]. Sturgeon herpesviruses can be transmitted horizontally from infected fish to naïve cohabitants and, in the case of AciHV-2, the virus was detected in gametes from wild broodstock, suggesting that vertical transmission is possible [8,14].

Diagnosis of sturgeon herpesvirus infections has occurred primarily through electron and light microscopy methods to visualize the virion architecture and host tissue pathology [7,8,12,14,15,16]. The histological changes typically include epithelial hyperplasia, intercellular oedema, infiltration of the epidermis by inflammatory cells and margination of the chromatin in enlarged nuclei [24,25]. The development of sturgeon cell lines permissive to sturgeon herpesvirus culture has facilitated the isolation of some of these viruses from infected tissues [7,12,14,15,28,29]. The high degree of host specificity that is characteristic of alloherpesviruses may lead to false negative cell culture test results—a factor that should be considered when designing a diagnostic pathway for reliable detection of these viruses [24,25]. Conventional PCR (cPCR) assays have been developed for diagnostic purposes or to conduct molecular analyses of sturgeon herpesvirus sequences [9,10,13,15,16,17,20,30]. Validated quantitative PCR tests for high-throughput detection of these viruses are not available. Next-generation sequencing (NGS) methods in concert with bioinformatic analyses have been used to identify Lake Sturgeon herpesviruses in the Great Lakes watershed [15,16].

We report the discovery of a novel sturgeon herpesvirus in juvenile Lake Sturgeon displaying epithelial skin lesions. Case histories are provided for two separate year classes of wild broodstock offspring that displayed clinical signs while they were being reared in the hatchery. Our molecular investigations of the virus, designated as Acipenser herpesvirus 3 (AciHV-3), and its taxonomic relationships provide evidence of a new virus species and genus in the *Alloherpesviridae* family.

## 2. Materials and Methods

### 2.1. Case History—Hatchery Case Study

**Figure 1:**
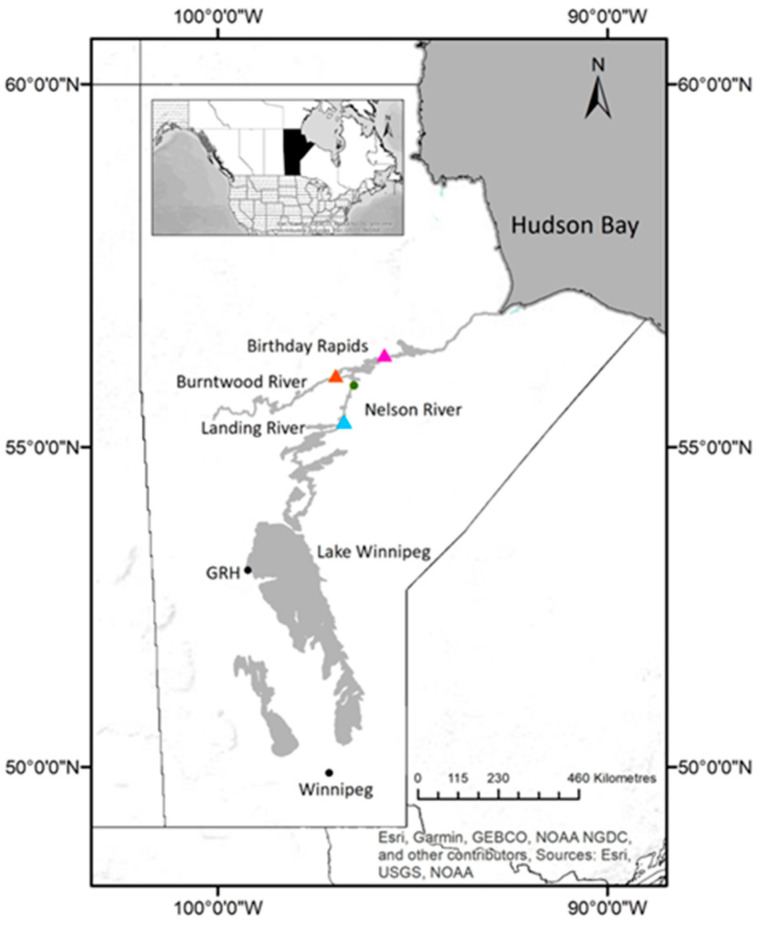
Location of spawning sites where gametes were collected from Lake Sturgeon *Acipenser fulvescens* and Grand Rapids Hatchery (GRH) where the progeny were cultured. Spawn collection sites are marked with colored triangles (blue, confluence of the Landing and Nelson Rivers; orange, First Rapids on the Burntwood River; pink, Birthday Rapids on the Nelson River). The green circle shows the site of the Kelsey Generating Station on the Nelson River. A map inset of North America provides the relative location of the province of Manitoba (black) in Canada (white). The map was created using ESRI [34] ArcGIS 10.8.1 software with publicly available CANVec Series shapefiles from Natural Resources Canada [35].

Spawn from wild adult Lake Sturgeon were collected in 2017 and 2018 from three spawning sites as part of conservation stocking programs that were initiated in 1994 and 2013 to supplement extirpated or at-risk populations of wild Lake Sturgeon in the upper Nelson River [31] and lower Nelson–Burntwood Rivers [32], respectively. For both years, collections of fertilized eggs from wild adults in the upper Nelson River (UNR; near the confluence of the Landing River), Burntwood River (BWR; downstream of First Rapids) or the lower Nelson River (LNR; downstream of Birthday Rapids) were reared at the Grand Rapids Hatchery (GRH; Grand Rapids, Manitoba) (Figure 1). The Burntwood River is a tributary of the Nelson River that flows from Lake Winnipeg to the Hudson Bay. The sturgeon populations that spawn on the Nelson River near the confluence of the Landing River are separated from the lower Nelson–Burntwood River populations by the Kelsey Generating Station (Figure 1). The lower Nelson and Burntwood River populations are genetically distinct (despite the absence of a barrier such as a hydroelectric generating station separating the two populations) [33]. This population structure means that each spawn site should only be stocked with progeny of broodstock originating from that site [33].

**Figure 2:**
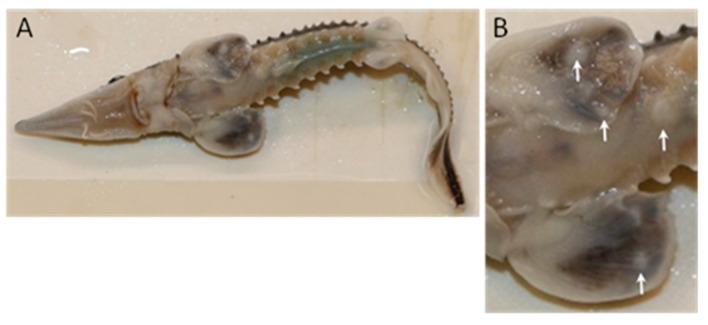
Epithelial lesions on age 0 Lake Sturgeon *Acipenser fulvescens* housed at Grand Rapids Hatchery. The offspring of wild broodstock from the lower Nelson River (2018 year class) displayed lesions ranging in diameter from 0.2 to 0.3 cm on the ventral surface of the abdomen and pectoral fins (**A**). Panel (**B**) is a zoom of the pectoral fin region of the same fish with lesions identified by the white arrows. Photo credit: Marek Tomczyk.

Epithelial lesions that resembled blisters appeared on the ventral surface of the pectoral fins and abdomen of age 0 progeny in both year classes of GRH Lake Sturgeon (Figure 2; see Section 3.1.1.). No unusual mortality or behavior was observed in these fish while they were housed at the hatchery.

#### 2.1.1. Grand Rapids Hatchery 2017

The 2017 year class (YC) of juvenile sturgeon at Grand Rapids Hatchery were the progeny of sexually mature wild broodstock collected from either the upper Nelson River or Burntwood River (Figure 1). The upper Nelson River offspring were produced using eggs from two females paired with milt from six males to create seven family groups. The progeny were housed at GRH in six tanks receiving recirculated well water. The water temperature was 16.65 ± 0.05 °C and fish weighed 3.12 ± 0.55 g (tail length (TL) 92.44 ± 5.41 mm) when skin growths were first observed by staff (28 August 2017) (Supplement S1). Eggs and milt from one female and four males from Burntwood River were paired to create two family groups and progeny were held in ten tanks, all of which received recirculated well water. The water temperature in these tanks on 28 August 2017 was 15.94 ± 0.16 °C and the fish weighed 1.96 ± 0.49 g (TL 79.97 ± 7.27 mm) (Supplement S1). The water quality parameters of temperature, dissolved CO_2_ and O_2_, nitrate, ammonia and pH were stable in all GRH tanks for the month (7–28 August) prior to the first appearance of skin lesions.

A subset of sturgeon from each tank was evaluated for lesions (i.e., presence/absence) on 31 August, 7, 18, 19 September 2017 (*n* = 30 per tank per timepoint, except 19 September, when all sturgeon in the hatchery were examined (*n* = 387 to 479 per tank)) (Table S5 in Supplement S1). Sturgeon from the upper Nelson River population were randomly selected from those displaying lesions, euthanized and submitted on 5 September 2017 for histology (*n* = 5) and bacteriology (*n* = 3) testing. diagnostic testing was conducted by the manitoba agriculture & resource development veterinary diagnostic services lab (Winnipeg, MB, Canada). sturgeon from one or both populations were euthanized on 5 september (*n* = 10 unr), 20 september (*n* = 15 unr) and 29 september (*n* = 12 unr; *n* = 24 bwr) and archived at −80 °C.

#### 2.1.2. Grand Rapids Hatchery 2018

Grand Rapids Hatchery received fertilized eggs in June 2018 from wild broodstock captured in the upper Nelson River (1 female, 4 males, 5 family groups) or the lower Nelson River (1 female, 4 males, 5 family groups) (Figure 1). Offspring from the two spawning sites were held in separate recirculating systems, each comprising six tanks receiving well water. Lesions were first observed on 5 October 2018. At that time, the average water temperature for the two systems was 10.3 ± 0.08 °C (upper Nelson River) and 8.5 ± 0.08 °C (lower Nelson River). The upper Nelson River juveniles weighed 5.11 ± 2.0 g (TL 105 ± 13 mm), whereas the lower Nelson River progeny were 3.88 ± 1.0 g (TL 97 ± 9.0 mm) (Supplement S1). With the exception of water temperature, no unusual fluctuations were observed in the water quality parameters for the month (September 2018) prior to the first observation of the lesions. The water temperature in the two recirculation systems was reduced to acclimate the fingerlings in preparation for their planned spring or fall stocking. The variation in water temperature was higher for the lower Nelson River population, which experienced a 9.3 °C drop in water temperature over 3 weeks from 17 september to 9 October 2018 (versus a reduction of 5.6 °C for the upper Nelson River population over the same period of time) (Figure S1 in Supplement S1).

Sturgeon from each tank were visually inspected for lesions (presence/absence) on 12 October (*n* = 30 per tank) and 30 November 2018 (*n* = 15 per tank) (Table S6 in Supplement S1). The upper Nelson River population was examined at subsequent timepoints, but the number of fish evaluated was not recorded. For the lower Nelson River population, a subset of sturgeon (*n* = 219) was isolated on 4 March 2019 and monitored for 10 weeks. These fish were selected because they either displayed lesions or showed potential evidence of previous lesions. Since staff removed three fish for sampling and three additional fish were added to this population, the total number fluctuated (*n* = 217 to 221) over the observation period. Lesion prevalence (presence/absence per fish) and density (number of lesions per fish) were recorded on 4, 13, 18, 26 March, 7, 22, 29 April and 7, 12 May 2019 (*n* = 5 to 84 per tank) (Tables S6 and S7 in Supplement S1). Sturgeon were randomly selected from the upper Nelson (*n* = 3) and lower Nelson (*n* = 3) River populations, euthanized and submitted on 17 October 2018 for bacteriology and histology testing. Diagnostic testing was conducted by the Manitoba Agriculture & Resource Development Veterinary Diagnostic Services Laboratory (Winnipeg, MB, Canada). Lower Nelson River sturgeon displaying lesions (*n* = 2) were euthanized on 16 April 2019 and processed for transmission electron microscopy by the National Center for Foreign Animal Disease laboratory (Winnipeg, MB, Canada).

#### 2.1.3. Statistical Analyses

Statistical analyses and visualization of lesion prevalence data were implemented in Stata/SE v.17.

### 2.2. Virology, Bacteriology, Histology and Electron Microscopy

#### 2.2.1. Viruses

The viruses and sequences included in this study are presented in Table 1.

#### 2.2.2. Virology—Hatchery Case Study

White Sturgeon skin (WSSK) [7], White Sturgeon spleen (WSS-2) [28], and Lake Sturgeon gill (LSGI) or gonad (LSGO) [29] cell monolayers were inoculated with single fish pools of kidney, brain, spleen, gill, snout, ventral abdominal skin and pectoral fins from symptomatic juvenile Lake Sturgeon reared at the GRH (*n* = 10, 2017YC upper Nelson River stock). Cells were grown in 25 or 75 cm^2^ flasks at 15 °C using minimal essential media with Hanks salts (MEMH) supplemented with 2 mM L-glutamine, 10% fetal bovine sera (FBS) and 0.02 M HEPES (MEMH10H) (Life Technologies, Burlington, ON, Canada). Antibiotic/Antimycotic (Gibco™. Stratford, ON, Canada) was added following virus adsorption. Clarified filtrates were prepared by diluting the same fish tissue pools 1:50 in MEMH10H containing 2× Antibiotic/Antimycotic, centrifuging at 1500× *g* for 2 min at 4 °C and filtering the supernatant through a 0.45-micron syringe filter. For virus adsorption, the medium was removed from the cell monolayer and clarified tissue homogenate or media (negative control) were added to each flask. After a 45 min incubation at 15 °C and the addition of 5 to 25 mL MEMH10H containing antibiotic/antimycotic, flasks were incubated at 15 °C and monitored for a cytopathic effect (CPE). Flasks with monolayers that displayed a putative cytopathic effect were placed at −80 °C until they were ready to be used.

#### 2.2.3. Bacteriology—Hatchery Case Study

Skin and kidney tissues from juvenile Lake Sturgeon of the 2017YC (*n* = 3, upper Nelson River cohort, 5 September 2017) and 2018YC (*n* = 3, upper Nelson River cohort; *n* = 3, lower Nelson River cohort; 17 October 2018) reared at the GRH were screened for the presence of bacteria by culture on blood agar and MacConkey agar [36]. Inoculated plates were held under aerobic conditions at 22 °C for 5 days and for 2 days at 35 °C. Gram staining [37] was performed on skin and gill tissue smears from the same fish. Skin tissue from the three 2017YC upper Nelson River sturgeon was tested for the presence of dermatophytes by culture on Sabouraud agar (with or without chloramphenicol (50 mg L^−1^)) [36] at 22 °C and 35 °C for 3 days. Gill wet mounts were also prepared for the same subset of fish.

#### 2.2.4. Histology—Hatchery Case Study

Whole juvenile Lake Sturgeon from the 2017YC (*n* = 5, upper Nelson River stock, 5 September 2017) and the 2018YC (*n* = 3, upper Nelson River stock; *n* = 3, lower Nelson River stock; 17 October 2018) were euthanized and placed in 95% ethanol or 10% buffered formalin prior to embedding portions of the fixed tissue in paraffin blocks. The tissues were sectioned to 5 μm thickness, deparaffinized, stained with hematoxylin and eosin (H&E) and examined for histopathology by light microscopy [38].

#### 2.2.5. Electron Microscopy—Hatchery Case Study

Skin growths (*n* = 14) were excised from 2 hatchery-reared juvenile Lake Sturgeon of the lower Nelson River population (2018YC) immediately following euthanasia with a lethal dose of tricaine methanesulfonate in April 2019. The tissue sections were initially placed in Karnovsky fixative, transferred to a modified Karnovsky fixative (2.5% glutaraldehyde + 2% paraformaldehyde in 0.1 M sodium phosphate buffer pH 7.2) for 2 h at 4 °C and then post-fixed for 1 h in 1% osmium tetroxide. Following dehydration in ethanol, the samples were infiltrated and embedded in an epoxy resin consisting of Araldite 505 and EMbed 812. Ultra-thin sections (90 nM) on 200 mesh copper grids were stained with 2% uranyl acetate and lead citrate.

Observations were made using a Phillips CM 120 transmission electron microscope at 80 kV. Digital images were captured using Hamamatsu ORCA HR camera and the AMT imaging system.

### 2.3. Viral Nucleic Acid

#### 2.3.1. DNA Synthesis and Plasmid Purification

The full-length major capsid protein (mcp) DNA sequence for LS AciHV-3 was synthesized and inserted into vector pJ248 (ATUM, Newark, CA, USA) for use as a molecular positive control sample. Plasmid DNA was transformed into MAX Efficiency^®^ *Escherichia coli* DH5α competent cells according to the manufacturer’s instructions (Invitrogen, Burlington, ON, Canada) and purified from *E. coli* overnight cultures using the QIAprep Spin Miniprep Kit as described by Qiagen (Toronto, ON, Canada).

#### 2.3.2. Nucleic Acid Extraction

Lake Sturgeon gonad cell monolayers displaying putative CPE after inoculation with pooled tissue homogenates from upper Nelson River Lake Sturgeon (2017YC) were used to prepare DNA for Illumina (San Diego, CA, USA) HiSeq Next-Generation Sequencing (see Section 2.2.2 for virology details). Flasks (*n* = 4 × 75 cm^2^) were removed from the −80 °C freezer and the contents thawed at 4 °C. A clarified cell suspension was created by scraping each monolayer into the supernatant, pipetting up and down several times to create an even suspension and pooling the contents of all 4 flasks prior to centrifugation at 2500× *g* for 15 min at 4 °C. The pellet was resuspended in 0.05% Trypsin-EDTA (Life Technologies, Burlington, ON, Canada) to dissociate any cell-associated virus, incubated at room temperature for 7 min and recombined with the clarified supernatant prior to repeating the centrifugation step. The virus was pelleted from the clarified supernatant following an 18 h incubation at 4 °C with 10% polyethylene glycol 8000 and centrifugation at 8000× *g* for 90 min at 4 °C. The pellet was resuspended in MEMH5 and the suspension was added to an equal volume of phenol:chloroform:isoamyl alcohol (25:24:1). The mixture was incubated at room temperature while gently rocking for 30 min and then centrifuged at 12,100× *g* for 20 min at room temperature. DNA in the aqueous phase was precipitated overnight at −80 °C with 0.3 M sodium acetate and 2 volumes of ice cold 100% ethanol, pelleted by centrifugation (12,100× *g*, 30 min, 4 °C), washed twice with 70% ethanol and resuspended in 10 mM Tris-HCl and 1 mM ethylenediaminetetraacetic acid pH 8.0.

**Table 1:** Virus taxa used to investigate Lake Sturgeon Acipenserid herpesvirus 3 systematics and genotype. BC, British Columbia; BWR, Burntwood River; GRH, Grand Rapids Hatchery; LNR, lower Nelson River; LS, Lake Sturgeon; MT, Montana; NB, New Brunswick; SnS, Shortnose Sturgeon; UCR, upper Columbia River; UNR: upper Nelson River; VIU, Vancouver Island University; WS, White Sturgeon; WSSK, WS skin cell line; WSS-2, WS spleen cell line; YC, year class.

Virus	Abbreviation	Accession Number(s)	Description of Use in Study ^d^
*Alloherpesviridae, Acivirus ^a^*			
** Acipenser herpesvirus 1**	AciHV-1		
Lake Sturgeon Black River	AciHV-1 200413-11TC	WAS29283.1, 84, 85, 87, 89	Systematics [15]
Lake Sturgeon Wolf River	AciHV-1 WR	UMM52714.1, 31, 41, 45, 47	Systematics [16]
White Sturgeon UC Davis	AciHV-1 UCD	UKB92883.1, 84, 85, 87, 88	Systematics [16]
** Acipenser herpesvirus 3 ^a^**	AciHV-3		
Atlantic Sturgeon AciHV-3	AciHV-3 JABEPO	JABEPO010010078.1 ^b^	Genotyping ^e^
Lake Sturgeon AciHV-3 63-201:210 ^c^	AciHV-3 UNR	OR242753, 54, 55, 56, 57	Systematics: 2017YC GRH UNR LS
Lake Sturgeon AciHV-3 63-207	AciHV-3 UNR 2017	OR242743	Genotyping: 2017YC GRH UNR LS
Lake Sturgeon AciHV-3 63-251	AciHV-3 BWR 2017	OR242744	Genotyping: 2017YC GRH BWR LS
Lake Sturgeon AciHV-3 71-206	AciHV-3 LNR 2018	OR242746	Genotyping: 2018YC GRH LNR LS
Lake Sturgeon AciHV-3 71-201	AciHV-3 UNR 2018	OR242745	Genotyping: 2018YC GRH UNR LS
Lake Sturgeon AciHV-3 LS gonad cell line	AciHV-3 LSGO	OR242742	Genotyping [29]
Pallid Sturgeon AciHV-3 MT 2008	AciHV-3 MT 2008	OR242752	Genotyping: DNA from R. Hedrick
Shortnose Sturgeon AciHV-3 54-1402	AciHV-3 NB 2015	OR242747	Genotyping: 2013YC SnS (NB hatchery)
Sterlet Sturgeon AciHV-3 Gen_M01	AciHV-3 Gen M01	VTUV01000924.1 ^b^	Systematics, genotyping ^e^
Sterlet Sturgeon AciHV-3 WHYD16114868_AA	AciHV-3 WHYD	SCEB01000000.1 ^b^	Systematics, genotyping ^e^
Sterlet Sturgeon AciHV-3 fAciRut3	AciHV-3 fAciRut3	OV754656^b^	Systematics, genotyping ^e^ [39]
White Sturgeon AciHV-3 63-1	AciHV-3 UCR 2017	OR242748	Genotyping: BC UCR wild WS (2017)
White Sturgeon AciHV-3 89-174	AciHV-3 VIU 2022	OR242749	Genotyping: VIU WS (2022)
White Sturgeon AciHV-3 WSS-2 cell line	AciHV-3 WSS-2	OR242751	Genotyping [28]
White Sturgeon AciHV-3 WSSK cell line	AciHV-3 WSSK	OR242750	Genotyping [7]
*Alloherpesviridae, Batravirus*			
Bufonid herpesvirus 1 FO1_2015	BfHV-1	YP_009552889.1, 891, 918, 933, 946	Systematics
Ranid herpesvirus I McKinnell	RaHV-1	YP_656709.1, 718, 727, 748, 750	Systematics
Ranid herpesvirus 2 Rafferty	RaHV-2	YP_656588.1, 596, 618, 637, 639	Systematics
Ranid herpesvirus 3 FO1_2015	RaHV-3	YP_009362382.1, 385, 398, 405, 419	Systematics
*Alloherpesviridae, Cyvirus*			
Anguillid herpesvirus 1 500138	AngHV-1	YP_003358175.1, 176, 194, 196, 243	Systematics
Cyprinid herpesvirus 1 NG-J1	CyHV-1	YP_007003734.1, 735, 741, 742, 755	Systematics
Cyprinid herpesvirus 2 ST-J1	CyHV-2	YP_007003890.1, 891, 897, 898, 911	Systematics
Cyprinid herpesvirus 3 F98-50	CyHV-3	YP_001096106.1, 107, 113, 114, 127	Systematics
*Alloherpesviridae, Ictavirus*			
**Acipenser herpesvirus 2**	AciHV-2		
Shortnose Sturgeon AciHV-2 NB_2015	AciHV-2 NB 2015	OR242758, 59, 60, 61, 62	Systematics
Snake River White Sturgeon herpesvirus	AciHV-2 SRWSHV	YP_009664566.1, 568, 569, 579, 599	Systematics
Ictalurivirus herpesvirus 1 Auburn 1	IcHV-1	NP_041116.1, 118, 119, 130, NP_041148.2	Systematics
Ictalurivirus herpesvirus 2 760/94	IcHV-2	YP_009447852.1, 853, 854, 864, 884	Systematics
Silurid herpesvirus 1 KRB14001	SiHV-1	AVP72202.1, 03, 04, 14, 65	Systematics
*Orthoherpesviridae, Alphaherpesvirinae, Simplexvirus*			
Human alphaherpesvirus 1 Strain 17	HSV-1	YP_009137079.1, 092, 093, 100, 105	Systematics
*Orthoherpesviridae, Gammaherpesvirinae, Rhadinovirus*			
Saimiriine gammaherpesvirus 2	SaHV-2	NP_040211.1, 219, 227, 228, 246	Systematics

^a^ Proposed classification for consideration by the International Committee on Taxonomy of Viruses.^b^ See Supplement S2 for sequence details.^c^ Samples used for next generation sequencing.^d^ Full length mcp DNA and protein sequences from viruses used in the systematics study were used to determine percent identity and similarity. ^e^ Sequence corresponding to the 493 bp sequence used for genotyping was extracted from the published sequence datasets for these samples.

AciHV-2 DNA for NGS analyses was isolated from WSS-2 cell monolayers displaying a cytopathic effect after inoculation with Shortnose Sturgeon AciHV-2 isolate 54–1401. WSS-2 cell monolayers were cultured as described in Section 2.2.2 and inoculated with a single fish homogenate of pooled pectoral fin, gill, operculum and snout tissue diluted 1:50 in MEMH2 containing 2x antibiotic/antimycotic (Gibco™, Stratford, ON, Canada). Five days later, the monolayers were harvested. A cell suspension was generated by detaching any residual monolayer using a cell scraper, pooling the contents of multiple flasks and storing aliquots of the material at −80 °C. The virus was partially purified from ten 1 mL aliquots after homogenization (two 5 mm stainless steel beads; Tissuelyzer (Qiagen, Toronto, ON, Canada); 3 Hz for 2 min), two sequential centrifugations (2500× *g*, 10 min, 4 °C), RNase A treatment of the clarified supernatant (30 μg/mL, 2.5 h, 37 °C) and another centrifugation to pellet the virus (20,627× *g*, 18 h, 4 °C). After resuspending the pelleted material in 180 μL Qiagen ATL buffer plus 20 μL Proteinase K (Qiagen, Toronto, ON, Canada), the suspension was incubated at 56 °C for 2 h and then DNA was extracted using the DNeasy Blood and Tissue Kit (Qiagen, Toronto, ON, Canada) as described by the manufacturer. Nucleic acid was quantified using a Nanodrop 8000 spectrophotometer (ThermoFisher Scientific, Mississauga, ON, Canada).

Pectoral fin tissue samples (*n* = 26) collected from age 0 Lake Sturgeon reared at Grand Rapids Hatchery in 2017 and 2018 were used to prepare DNA for genotyping the presumptive AciHV-3 isolates. DNA was also extracted from pectoral fin tissue samples from Shortnose Sturgeon (Charlo, NB, Canada) and White Sturgeon (Nanaimo, BC, Canada; upper Columbia River, BC, Canada) (Table 1). Samples were processed using the MagMax™ CORE Nucleic Acid Purification Kit (Applied Biosystems™, Mississauga, ON, Canada) on the KingFisher Flex Purification System (ThermoFisher, Mississauga, ON, Canada) or the DNeasy Blood and Tissue Kit (Qiagen, Toronto, ON, Canada). Each fin tissue sample was trimmed to ≤25 mg, placed in ATL buffer (Qiagen, Toronto, ON, Canada) containing 2 mg/mL Proteinase K (Qiagen, Toronto, ON, Canada) and homogenized using the TissueLyser II (Qiagen, Toronto, ON, Canada) operated twice at 25 Hz for 2 min. Tissue homogenates were incubated in an Eppendorf (Mississauga, ON, Canada) ThermoMixer^®^ (18 h, 300 rpm, 56 °C) and centrifuged (5 min, 21,000× *g*, room temperature) prior to extracting DNA from the clarified supernatant as per each manufacturer’s instructions. The extracted DNA was measured on the NanoDrop 8000 spectrophotometer (ThermoFisher Scientific, Mississauga, ON, Canada) or Qubit 4.0 fluorometer using the 1× dsDNA High-Sensitivity Assay Kit (Invitrogen, Burlington, ON, Canada). A maximum of 500 ng of DNA was added to each cPCR reaction.

#### 2.3.3. PCR and Sanger Sequencing

A conventional PCR assay, hereafter GCmcp, was designed to amplify a 536 bp portion of the AciHV-3 major capsid protein DNA sequence (bp 1854–2389 of accession number OR242757). The target region was identified from DNA sequence alignments of full-length mcp gene sequences from Lake Sturgeon AciHV-3 (this study) and Sterlet Sturgeon AciHV-3 (Table 1). The alignment revealed a number of single nucleotide polymorphisms in the Sterlet Sturgeon sequence relative to that of the Lake Sturgeon. Candidate primer sequences were identified using the PCR primer design tool in the SeqBuilder Pro module of Lasergene v.17 (DNASTAR, Madison, WI, USA). The GCmcp assay primers were LSBVgenomcpF1 (5′ CCATTTCAAAGACGCGGCCAT 3′) and LSBVgenomcpR1 (5′ TGTAGATGCCCAGGCCCTTCAG 3′). Each 25 μL reaction consisted of 1× Ampli-Taq Gold universal master mix, 200 nM of each primer, 200 μM of each deoxyribonucleotide triphosphate, 1.5 mM MgCl_2_, 1.25 units Ampli-Taq Gold DNA polymerase (Applied Biosystems, Mississauga, ON, Canada) and 100 to 1500 ng DNA. The 536 bp product was generated after 1 cycle of 95 °C for 5 min, 40 cycles of 95 °C for 30 s, 59 °C for 30 s and 72 °C for 60 s, followed by 1 cycle of 72 °C for 10 min. The purity and size of PCR products were visualized in 1% agarose gels by gel electrophoresis. Amplicons were purified from the agarose gel using the QIAquick Gel Extraction Kit (Qiagen, Toronto, ON, Canada) as instructed by the manufacturer. The purified DNA was sequenced using the Sanger method [40] by the Sanger Sequencing Facility at the Hospital for Sick Children (Toronto, ON, Canada). The results were analyzed using BioEdit v7.0.5.3 software [41]. The DNA sequence used for AciHV-3 genotyping was 493 bp (no primer sequences).

### 2.4. Next-Generation Sequencing, Assembly and Annotation

Library preparation, next-generation sequencing and sequence assembly were performed by The Center for Applied Genomics at The Hospital for Sick Children (Toronto, ON, Canada).

#### 2.4.1. NGS and Assembly of the AciHV-2 Dataset

Libraries for two DNA samples isolated from WSS-2 cell monolayers infected with AciHV-2 isolate 54–1401 were prepared using the Illumina TruSeq Nano DNA and Nextera XT DNA Library Prep kits. Sequencing was performed on the HiSeq 2500 Platform (Illumina, San Diego, CA, USA) with 2 × 125 cycles in paired-end mode and generated 27 or 53 million read pairs from each respective library. Sequence data quality was evaluated using FastQC v.0.11.4 (Babraham Bioinformatics). Illumina adapter sequences and low-quality ends (Phred score < 20) were trimmed using Trim Galore v.0.4.1 (Babraham Bioinformatics) and sequence quality was rechecked using FastQC. Reads below 40 bp in length were excluded from further analyses. The trimmed reads were screened using FastQ Screen v.0.7.0 (Babraham Bioinformatics) against the following alloherpesvirus reference genomes (National Center for Biotechnology Information (NCBI) Genbank accession number (virus, sequence length)): NC_001493.2 (IcHV-1, 134,226 bp), NC_008211.1 (RaHV-1, 220,859 bp), NC_008210.1 (RaHV-2, 231,801 bp), NC_009127.1 (CyHV-3, 295,146 bp), NC_013668.3 (AngHV-1, 248,526 bp), FJ815289.2 (AciHV-2 SRWSHV, partial genome of 66,037 bp) and FJ815290.1 (IcHV-2, partial genome of 7982 bp).

De novo assembly of the trimmed reads from each library and reads pooled from both libraries was performed using SPAdes v3.9.0 [42] in meta mode [43]. The program was run with MisMatchCorrector turned on and k-mer lengths of 21, 33, 55, 77 and 99. The largest contigs assembled were 26 and 118 kbp for the individual libraries and 126 kbp for the pooled reads.

#### 2.4.2. NGS and Assembly of the AciHV-3 Dataset

An Illumina (San Diego, CA, USA) TruSeq DNA PCR-free library was prepared for DNA extracted from Lake Sturgeon gonad cell monolayers displaying putative CPE after inoculation with tissue homogenates from the upper Nelson River Lake Sturgeon (see Section 2.3.2 for details). Sequencing was performed on the HiSeqX platform (Illumina, San Diego, CA, USA), with 2× 150 cycles in paired-end mode, and resulted in 121 million read pairs. Sequence data quality was evaluated using FastQC v.0.11.9 (Babraham Bioinformatics). Trim Galore v.0.4.4 (Babraham Bioinformatics) was used to remove the first 10 bp of each paired read as well as trim the Illumina adaptors and low-quality ends (Phred score < 20). The trimmed data quality was checked using FastQC. Reads less than 40 bp in length were excluded from assembly. The final dataset consisted of 120.6 million reads.

De novo assembly of the trimmed data set was performed using SPAdes v3.13.0 [42] with MisMatchCorrector turned on and k-mer lengths of 21, 33, 55, 77 and 99. The reads were not normalized prior to assembly. The largest contig assembled was 144 kbp in length with a k-mer coverage of 72.

#### 2.4.3. Annotation of the AciHV-2 and AciHV-3 Datasets

Putatively functional open reading frames (ORF) were delineated on the contig using the Seqman Pro module of Lasergene v.17 (DNASTAR, Madison, WI, USA), GenemarkS [44] and Genome Annotation Transfer Utility [45] software. An ORF was defined as a contiguous nucleotide sequence having a translation start and end codon and a length of >50 bp. The NCBI BLASTP program [46] was used to identify Lake Sturgeon AciHV-3 and Shortnose Sturgeon AciHV-2 homologs of alloherpesvirus core proteins encoded by the contigs.

### 2.5. Sequence Similarity, Phylogenetics and Genotyping Analyses

#### 2.5.1. Identity Matrix for Alloherpesvirus Major Capsid Protein Sequences

The percentage amino acid identity of mcp sequences was determined using the MegAlign Pro module of Lasergene v.17 (DNASTAR, Madison, WI, USA).

#### 2.5.2. Phylogenetic Analyses

Phylogenetic analyses for identifying genotypes and exploring the evolutionary relationships of AciHV-3 isolates to other alloherpesviruses were conducted using the virus sequences described in Table 1 (see also Supplement S2). The viruses were classified based on the 2022 virus taxonomy published by the International Committee on Taxonomy of Viruses [21] and included representatives from the *Alloherpesviridae* genera *Ictavirus*, *Batravirus* and *Cyvirus*.

AciHV-3 systematics was explored using five core alloherpesvirus proteins: capsid maturation protein, capsid triplex subunit 2, DNA polymerase catalytic subunit, helicase primase helicase subunit and the major capsid protein. Full-length amino acid sequences corresponding to these proteins were retrieved for alloherpesviruses and herpesviruses from the NCBI Genbank protein database and from the SnS AciHV-2 and LS AciHV-3 sequence datasets generated in this study (Table 1; Supplement S2).

Genotyping of the AciHV-3 isolates was conducted with a 493 bp DNA fragment of the mcp gene. The sequences were determined from PCR products amplified from various templates using the GCmcp assay described in Section 2.3.3 or from homologous sequences found in the NCBI Genbank nucleotide database (Table 1; Supplement S3).

Phylogenetic trees were reconstructed using individual DNA or concatenated protein sequences aligned with ClustalX2 (default parameters) [47]. The model selection option of Molecular Evolutionary Genetics Analysis (MEGA v7 or 11) software [48] was used to identify suitable nucleotide and amino acid substitution models based on their relative Bayesian information criterion score [49]. Phylogenies were generated in a Bayesian framework using Markov chain Monte Carlo methods with the BEAST software package (v1.10.4) [50]. Protein sequences were initially analyzed under the Yule model [51,52] (amino acid substitution prior: BLOSUM62 [53]; site heterogeneity prior: gamma and invariant) and a strict clock. The results were compared with those from the best-fit model identified in MEGA (v7 or 11) [48], and the tree with the lowest posterior mean value was selected. For the nucleotide sequences, analyses were conducted under the coalescent model [54] with HKY [55] as the nucleotide substitution prior and a strict clock prior. The results after 10 to 100 million generations were analyzed, annotated and displayed with Tracer v1.7.1 [56], TreeAnnotator v1.10.4 (BEAST v1.10.4) and FigTree v1.4.4 [57]. MCMC convergence diagnostics included estimated sample size values >200 [58] and visual confirmation of an appropriate MCMC trace topology.

## 3. Results

### 3.1. Lesion Gross Pathology and Prevalence

#### 3.1.1. Gross Pathology

The age 0 Lake Sturgeon at GRH displayed lesions that were macroscopically similar in both year class populations. Lesions were focal or multifocal to coalescing and resembled smooth, circular or irregularly shaped blisters (Figure 2). They were only observed on the ventral surface of the fish, either on the pectoral fins or mid-abdominal integument. They were elevated and pale relative to the surrounding skin tissue, and the skin surface appeared intact. Gross clinical signs were not evident in other organs including the gills, brain, eyes, operculum, heart, liver, kidney, stomach, spleen, pancreas and intestine.

#### 3.1.2. Lesion Prevalence and Density

**Figure 3:**
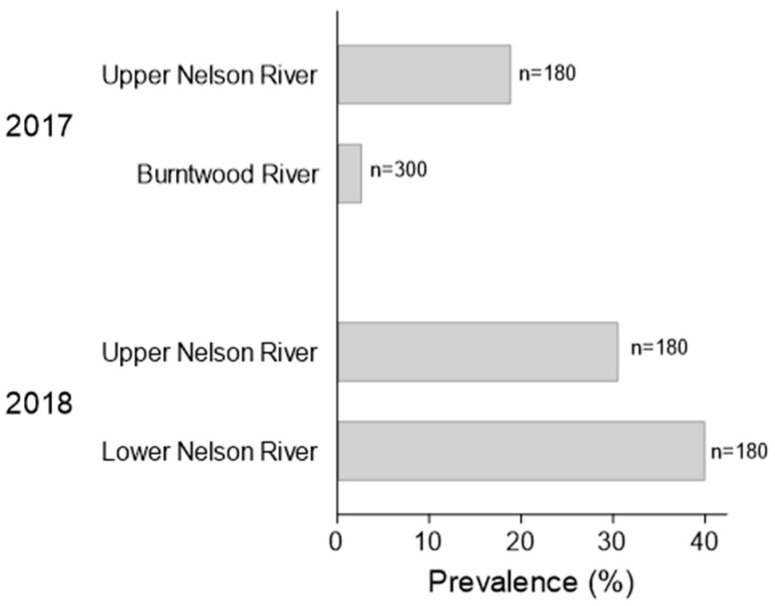
Prevalence of lesions upon their discovery in age 0 Lake Sturgeon *Acipenser fulvescens* housed at Grand Rapids Hatchery in 2017 and 2018. The sturgeon were progeny of wild broodstock from two rivers in northern Manitoba (Canada). The lesions were initially discovered in the 2017 year class (YC) on 28 August 2017, and prevalence levels were determined by evaluating the ventral surface of sturgeon in the hatchery on 31 August 2017 (*n* = 180 or 300 fish (6–10 tanks, 30 fish per tank)). For the 2018YC, lesion prevalence was determined on 12 October 2018, by inspection of 180 sturgeon randomly selected from each population one week after lesions were first observed (6 tanks, 30 fish per tank).

**Figure 4:**
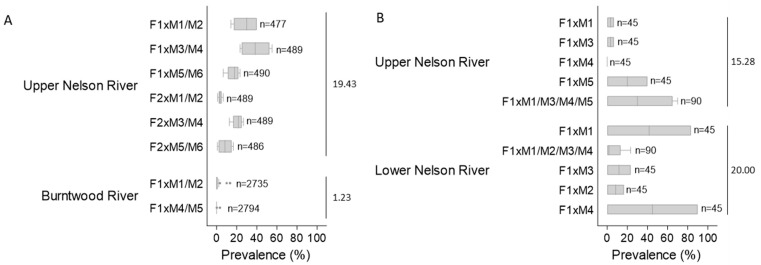
Lesion prevalence in families of juvenile Lake Sturgeon *Acipenser fulvescens* reared at Grand Rapids Hatchery in 2017 (**A**) and 2018 (**B**). Lesion prevalence in the 2017 year class (YC) was determined for each family on four sampling dates in August and September 2017; for the 2018YC, prevalence was determined for each family on two sampling dates in October and November 2018 (Tables S5 and S6 in Supplement S1). The vertical line within each horizontal box represents the median, each box encompasses 50% of the observations, the upper and lower whisker limits are 1.5 times the box length, grey circles beyond the whiskers are outliers and the number of fish evaluated is shown to the right of each box. The mean population prevalence is provided on the far right of each plot.

The average prevalence of lesions three days after their initial discovery in the 2017YC of sturgeon at the hatchery was 18.89 ± 12% for the upper Nelson River stock; for the Burntwood River population it was 2.67 ± 6% (31 August 2017; Figure 3). Lesion prevalence across families of upper Nelson River sturgeon ranged from 1 to 55% over the four sampling dates in 2017, with a median intra-family prevalence of 3 to 38% and a mean population prevalence of 19.43 ± 15% (Figure 4A). Over the same timeframe, lesion prevalence in the Burntwood River tanks varied from 0 to 13% and for both families; the median prevalence was 0%, with a population mean of 1.23 ± 3% (Figure 4A).

**Figure 5:**
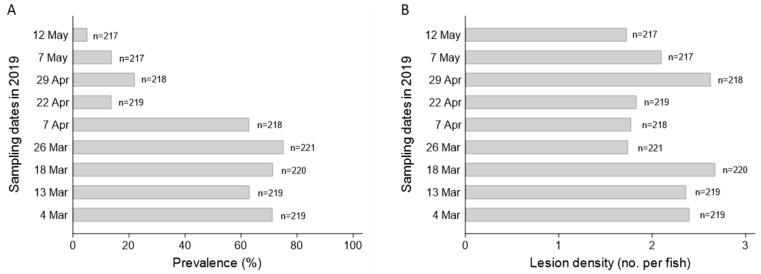
Timecourse of mean lesion prevalence (**A**) and density (**B**) in progeny of wild Lake Sturgeon *Acipenser fulvescens* broodstock from the lower Nelson River (2018 year class). At each timepoint, all sturgeon from the isolated subset (*n* = 217–221) were visually inspected for the presence and number of epithelial lesions.

The average prevalence of lesions one week after their initial observation in the 2018YC of sturgeon at the hatchery was 30.56 ± 30% for the upper Nelson River group and 40.00 ± 37% for the lower Nelson River population (12 October 2018; Figure 3). Between mid-October and the end of November 2018, lesion prevalence across tanks housing the upper Nelson River population ranged from 0 to 70%; for the lower Nelson River group, it varied between 0 and 90% (Figure 4B). The median prevalence across families was 0 to 30% (upper Nelson River) or 2 to 45% (lower Nelson River), and the mean prevalence within each population was 15.28 ± 26% (upper Nelson River) and 20.00 ± 32% (lower Nelson River) (Figure 4B). By 30 November, lesion prevalence in the upper and lower Nelson River populations was 0%. In the subset of lower Nelson River sturgeon isolated for long-term monitoring, lesion prevalence was above 60% between March and April 2019, after which the prevalence started to decrease (Figure 5A; Table S6 in Supplement S1). By 12 May 2019, the prevalence was at 5% (Figure 5A). The lesion density in the same group varied between fish (*n* = 1 to 14 lesions per fish), but the average intra-fish lesion density in the isolated population remained consistent between sampling dates (*n* = 1.7 to 2.7) (Figure 5B; Table S7 in Supplement S1). Lesions that were once evident eventually regressed. In some cases, visible scars and/or petechial hemorrhaging were evident at the site of the regressed lesion.

Within each of the four Lake Sturgeon populations reared at the GRH in 2017 and 2018, inter-family lesion prevalence was not significantly different except for family crosses of the upper Nelson River broodstock in 2017 (*p* < 0.05) (Figure 4). Inter-population lesion prevalence was significantly different for all populations relative to the Burntwood River group (*p* < 0.05) (Figure 4). No other significant differences were identified.

#### 3.1.3. Bacteriology and Virology

Bacterial culture from skin or kidney swabs revealed low levels of bacteria, including *Rhizobium radiobacter*, *Cellulomonas* sp., *Chryseobacterium indolegenes*, *Chryseobacterium ureilylticum*, *Bacillus* sp., *Sphingomonas paucimobilis*, *Plesiomonas shigelloides*, *Yokenella regensburgei*, *Aeromonas* sp. and *Vibrio metschnikovii*. These bacteria were not considered to be clinically important as they were not detected in all fish, were present in low levels and are known to be ubiquitous in aquatic environments.

**Figure 6:**
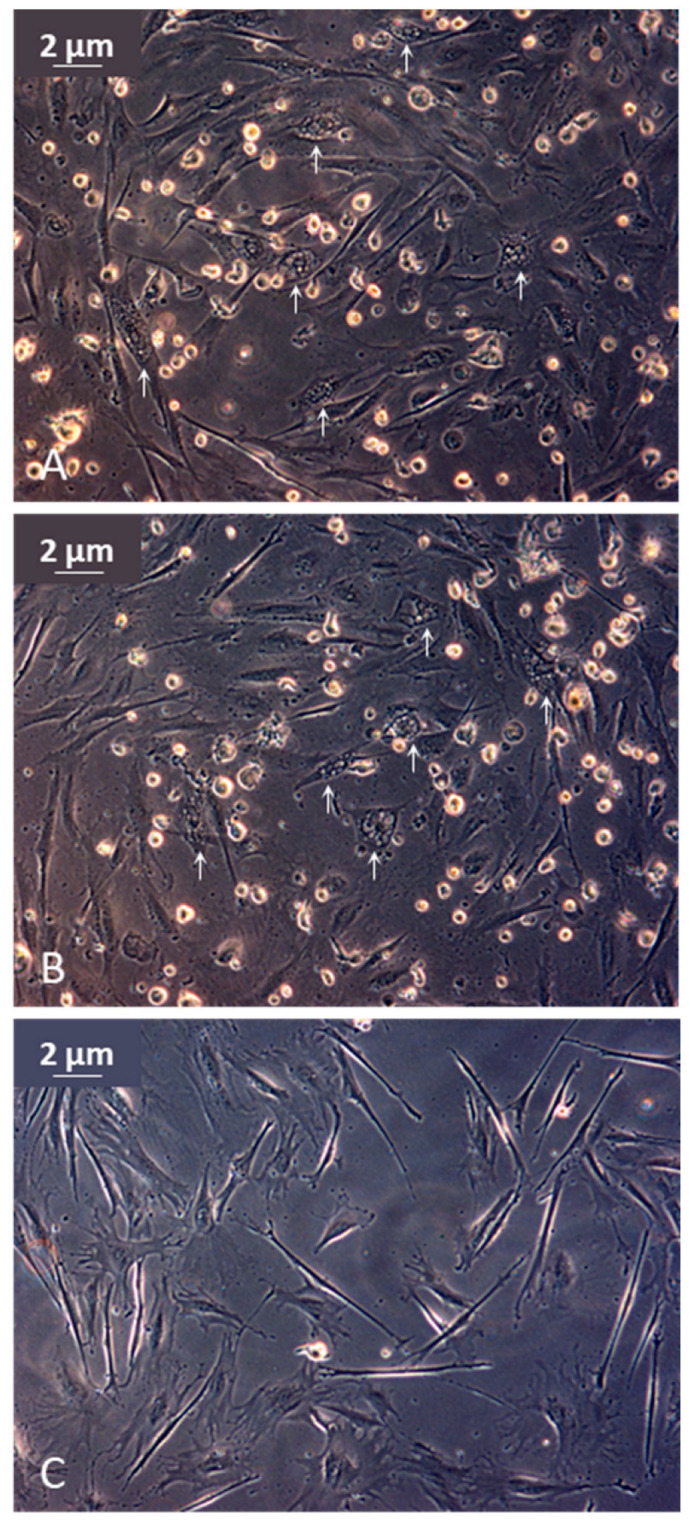
Light photomicrograph of Lake Sturgeon gonad cell monolayers infected with tissue homogenates from juvenile upper Nelson River Lake Sturgeon *Acipenser fulvescens* displaying epithelial lesions while housed at Grand Rapids Hatchery in 2017. (**A**,**B**) Eleven days after inoculation, infected monolayers displayed putative cytopathic effects that included free floating, rounded refractile cells and cytoplasmic vacuolization (white arrows). (**C**) Mock-infected normal monolayer.

Lake Sturgeon gonad cell monolayers inoculated with tissue homogenates from lesion-positive fish displayed cellular changes that were interpreted as a putative cytopathic effect. A progression of cellular changes was observed between 4- and 10-days post-infection (dpi). Hypertrophic cells were initially observed in the monolayer at 4 dpi and rounded refractile cells were present in the supernatant. By 6 dpi, enlarged, irregularly shaped cells containing numerous cytoplasmic vacuoles were observed throughout the monolayer, with more detached cells present in the supernatant (Figure 6A,B). The number of cells displaying vacuolization, the density of vacuoles within a cell and the number of detached cells increased between 4 and 10 dpi. The cytopathic effect did not progress further and complete destruction of the monolayer was not observed by 16 dpi when the monolayers were harvested. These cellular changes were diminished or not evident following blind passage of the supernatant onto a new monolayer of LSGO cells. The negative control LSGO cell monolayers inoculated with sterile media did not display these changes (Figure 6C). Noticeable CPE was not observed when the same samples from lesion-positive fish were inoculated onto WSSK, WSS-2 or LSGI cell monolayers.

#### 3.1.4. Histology and Electron Microscopy

**Figure 7:**
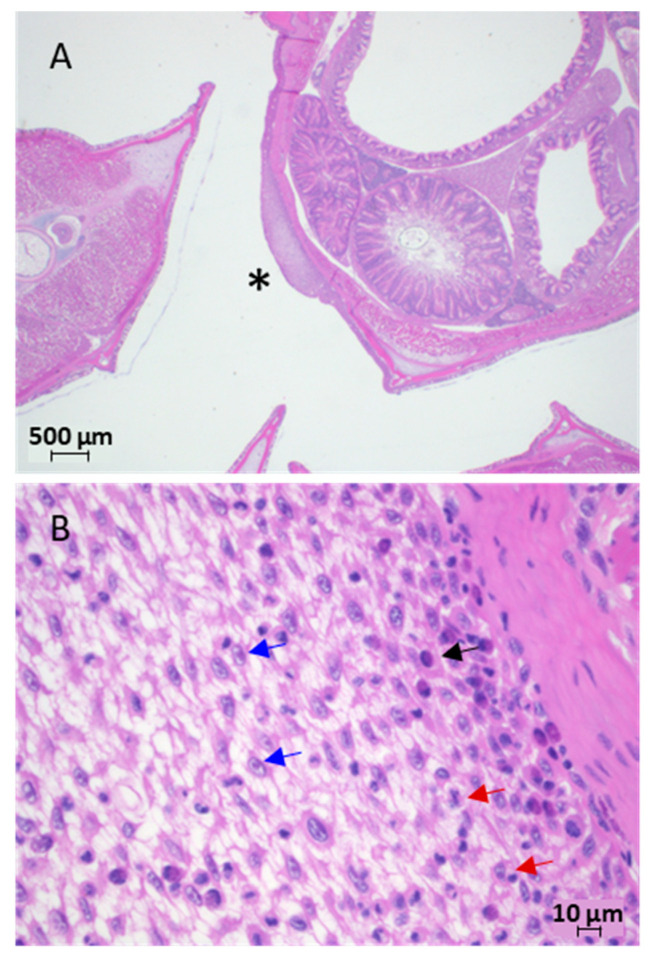
Light photomicrograph of hematoxylin-and-eosin-stained tissue from juvenile Lake Sturgeon *Acipenser fulvescens* displaying epithelial lesions while housed at Grand Rapids Hatchery. (**A**) Cross section through body near foregut of sturgeon from the lower Nelson River (2018 year class). (**B**) The focal area of epidermal oedema and hyperplasia marked with an asterisk in panel A is magnified to show prominent bridging between cells, chromatin margination in the nucleus (blue arrows) as well as monocytes (black arrow) and macrophages (red arrows) located deeper within the epidermal layer.

Histological evaluation of H&E-stained tissue sections of skin and fin lesions from juvenile Lake Sturgeon revealed focal areas of epidermal hyperplasia that were two to three times thicker than the adjacent epidermis (Figure 7A). The proliferative epidermitis was characterized by oedema, sub-epidermal lymphatic ectasia, mild to moderate leukocytic transpedesis of lymphocytes, monocytes and plasma cells and epithelial cells, some of which contained cytoplasmic vacuoles, an enlarged nucleus and/or margination of the chromatin (Figure 7B). This pathology was not found in histological sections of other organs from the same fish (i.e., brain, eyes, operculum, gills, mouth, barbels, heart, liver, stomach, pancreas, spleen or kidney).

Virus was not observed in the tissue lesions by transmission electron microscopy, despite extensive analyses.

### 3.2. AciHV Contigs Encode Alloherpesvirus Core Proteins

The largest contig assembled from the Illumina sequence data for AciHV-3 UNR was 144 kb, whereas that for AciHV-2 NB 2015 was 126 kb. BLASTP analyses of the amino acid sequences encoded by the predicted open reading frames revealed homologs of core proteins conserved among alloherpesviruses. The gene sequences of five core proteins were delineated from each dataset and have been deposited in the NCBI Genbank database under the following gene names and accession numbers (SnS AciHV-2, LS AciHV-3): capsid maturation protease (OR242758, OR242753), capsid triplex subunit 2 (OR242759, OR242754), DNA polymerase catalytic subunit (OR242760, OR242755), helicase primase helicase subunit (OR242761, OR242756) and major capsid protein (OR242762, OR242757) (Table 1, Supplement S2).

### 3.3. AciHV-3 Major Capsid Protein Sequences Are Highly Conserved

Comparison of the full-length mcp sequences from the AciHV-3 isolates showed that the DNA sequences were 99.5–100% identical (Table S14 in Supplement S3) and the protein sequences shared 99.6–100% similarity and 99–100% identity (Table 2). Similar comparative analyses conducted with the mcp sequences from AciHV-3 and AciHV-1 isolates revealed relatively lower levels of conservation for the DNA (55–59% identity) and the protein (31–32% identity; 48% similarity) sequences. The AciHV-3 and AciHV-2 isolates displayed the lowest level of mcp conservation amongst the sturgeon herpesviruses, with 40–41% identity for the DNA sequence (Table S14 in Supplement S3) and identity and similarity ranging from 24 to 25% and 43%, respectively, for the protein sequence (Table 2). Pairwise analyses of the AciHV-3 mcp sequences with those of alloherpesviruses from the *Batravirus* or *Cyvirus* genera produced estimates of 32–40% similarity, with identity ranging from 18 to 25% for protein and 40 to 52% for DNA (Table 2, Table S14 in Supplement S3).

### 3.4. AciHV-3 Is a New Species and Member of a New Alloherpesvirus Genus

**Figure 8:**
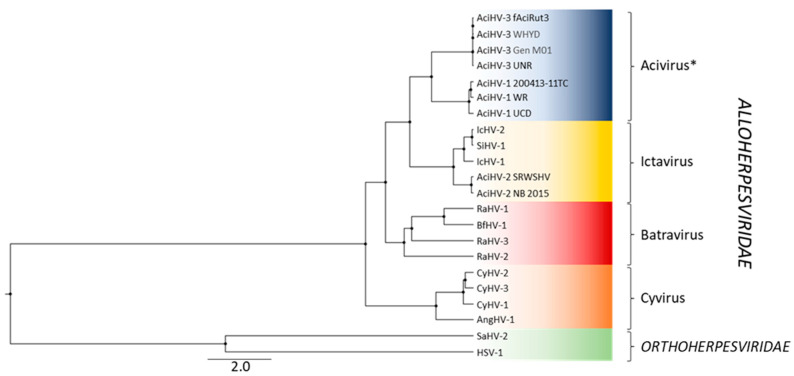
Phylogenetic tree of concatenated amino acid sequences of five core herpesvirus proteins from alloherpesviruses (capsid maturation protease, capsid triplex subunit 2, DNA polymerase catalytic subunit, helicase primase helicase subunit, major capsid protein). Concatenated full-length protein sequences were used to generate the tree. Nodes are marked with a black circle of fixed size and all node posterior probabilities are 1.0. Virus abbreviations are described in Table 1. The AciHV-3 isolate from this study is labelled as AciHV-3 UNR. The *Alloherpesviridae* genera are provided to the right of the tree. * The genus name Acivirus is proposed for the clade comprised of the unclassified AciHV-1 and new AciHV-3 taxa.

Phylogenetic reconstruction performed with a concatenation of five core alloherpesvirus proteins showed that AciHV-3 from Lake Sturgeon and Sterlet Sturgeon clustered together as a monophyletic sister clade to the AciHV-1 lineage (posterior probability of 1.0) (Figure 8). These two monophyletic clades descended from a strongly supported ancestral node (posterior probability of 1.0) that shared a more basal common ancestor with the ictaviruses (posterior probability of 1.0). Within that clade, North American isolates of AciHV-2 from Shortnose Sturgeon and White Sturgeon descended from a common ancestor shared with catfish alloherpesviruses (posterior probability of 1.0) (Figure 8). The batra- and cyviruses descended from the two most basal ancestral nodes of the *Alloherpesviridae* clade. The AciHV-3-AciHV-1 clade likely represents a new alloherpesvirus genus that we have designated as Acivirus.

### 3.5. AciHV-3 Is Present in Other Sturgeon Species

**Figure 9:**
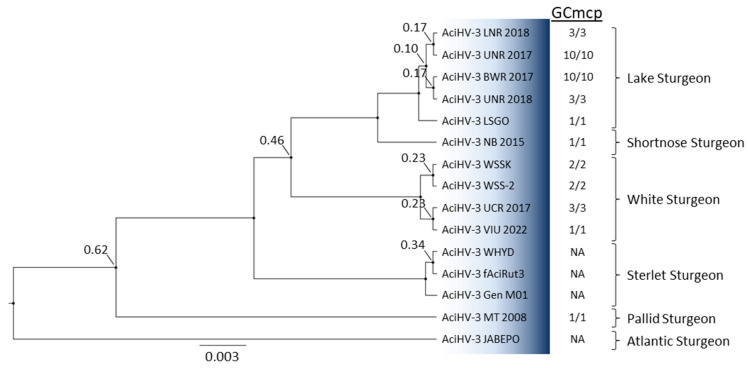
Bayesian phylogeny of the 493 bp major capsid protein DNA sequence from AciHV-3 taxa. Nodes are marked with a black circle of fixed size and node posterior probabilities < 0.994 are provided. Virus abbreviations are described in Table 1. Diagnostic test results (no. of positive/no. tested) obtained with the GCmcp genotyping PCR assay are shown to the right of the tree. PCR testing was not applicable (NA) to those taxa whose sequences were obtained from the Genbank database. The host origin of each sequence is provided on the far right of the figure.

The GCmcp cPCR assay amplified a product of the expected size (536 bp) from DNA samples extracted from the 2017YC (UNR *n* = 10; BWR *n* = 10) and 2018YC (UNR *n* = 3; LNR *n* = 3) of Lake Sturgeon displaying clinical signs at Grand Rapids Hatchery (Figure 9). DNA sequence analysis of the amplicon produced from at least one representative per river per year class confirmed that the correct sequence had been amplified. Amplicons of the expected size and sequence were also amplified from Shortnose Sturgeon (Charlo, NB, Canada), Pallid Sturgeon *Scaphirhynchus albus* (Miles, City, MT, USA) and White Sturgeon (Nanaimo, BC, Canada; upper Columbia River, BC, Canada). DNA isolated from naïve sturgeon cell lines unexpectedly produced a product of the predicted size and sequence from our Lake Sturgeon cell lines, as well as WSSK and WSS-2 cell lines from two different laboratories. A search of published sturgeon sequence datasets identified homologous sequences from Sterlet Sturgeon and Atlantic Sturgeon *A. oxyrhynchus oxyrhynchus* (Table 1). Pairwise comparison of the 493 bp DNA sequence from each of these sources revealed that the Lake Sturgeon AciHV-3 isolates shared 100% identity with each other and >95% identity with AciHV-3 isolates from Shortnose Sturgeon (99.4%), White Sturgeon (97.8%, BC White Sturgeon; 98%, White Sturgeon cell lines), Sterlet Sturgeon (97.8%), Pallid Sturgeon (96.6%) and Atlantic Sturgeon (95.7%). The single nucleotide polymorphisms resulted in synonymous and non-synonymous changes to the coding amino acids relative to the Lake Sturgeon genotyping sequence data (Tables S15 and S16 in Supplement S3). The nonsynonymous changes occur primarily in two regions of the mcp gene sequence used for genotyping (bp 1885–1971 (5/15 synonymous SNPs) and 2281–2363 (2/8 synonymous SNPs)) and are separated by a more conserved region with primarily synonymous changes (mcp bp 2035–2262 (11/13 synonymous SNPs); Tables S15 and S16 in Supplement S3)). Phylogenetic analysis performed with the 493 bp of the mcp gene from six sturgeon species showed that the AciHV-3 isolates clustered in monophyletic lineages according to the host (Figure 9).

## 4. Discussion

A new alloherpesvirus, with the proposed name of AciHV-3, was found in two consecutive year classes of hatchery-reared Lake Sturgeon displaying proliferative epithelial lesions. Although virus particles were not observed in the lesions via ultrastructural microscopy, an *Alloherpesviridae*-specific sequence was identified by NGS analysis of DNA from cultured cells inoculated with tissue homogenates from sturgeon displaying lesions and by Sanger sequencing of DNA amplified from lesion-positive sturgeon. Phylogenetic analyses revealed a distinct lineage for sturgeon herpesviruses that share a common evolutionary past with AciHV-1 taxa. AciHV-3 was identified in at least six sturgeon species.

Additional work will be required to establish disease causality between the presence of AciHV-3 virions or DNA and the epidermal cell proliferation observed in Lake Sturgeon at Grand Rapids Hatchery. The location of the lesions on the ventral surface of individual fish made it difficult to diagnose when the lesions first appeared. If the lesions sampled for transmission electron microscopy were from later in the virus infection cycle, it may explain why virus particles were not observed by microscopy. Notably, virus particles have yet to be found in Salmonid herpesvirus type 2-induced epidermal papilloma cells, a finding that highlights the difficulty associated with using electron microscopy as a diagnostic method [59,60,61]. Putative CPE was detected in the LSGO cell line inoculated with tissue homogenates from the 2017YC of upper Nelson River Lake Sturgeon displaying lesions. Although culture deterioration was observed (i.e., the appearance of vacuoles), the cell line did not appear to be lytically permissive to AciHV-3. The cytopathic effect was not consistent with the syncytia associated with AciHV-1 CPE or the grape-like clusters produced by AciHV-2 in White Sturgeon cell lines [7,8]. The epithelial lesions observed in both year classes of GRH Lake Sturgeon were pathognomonic in gross appearance, histopathology and tissue tropism with other fish herpesviruses. Samples from symptomatic fish tested positive with the AciHV-3-mcp-specific genotyping PCR assay. No other virus sequences were detected by NGS. Collectively, these observations support an association between the sturgeon herpesvirus sequence detected by NGS and Sanger sequencing and the lesions displayed by the Lake Sturgeon reared at the hatchery.

Herpesvirus disease can manifest as (1) an acute infection resulting in mortality, (2) a latent infection during which the non-replicating virus can be difficult to detect or (3) a recrudescent virus expression characterized by epidermal cell proliferation [24,25]. Our observations with AciHV-3 are consistent with the virus displaying a recrudescent type of infection, in that no unusual mortality occurred in Lake Sturgeon populations housed at the hatchery in 2017 and 2018, virus DNA was detected in all fish displaying lesions and focal epithelial hyperplasia of the integument was the primary clinical sign. All other organs appeared normal. These findings then imply the previous exposure of these age 0 fish to the virus, recovery and then establishment of a persistent infection. The fish were progeny of wild broodstock whose gametes were collected in the field and the eggs fertilized prior to transport. Virus exposure may have occurred as a result of horizontal transmission during sample collection, egg fertilization, transport or hatchery rearing. However, a vertical transmission pathway seems more likely, particularly given that there was no evidence of a previous infection (i.e., mortality) and the sturgeon were only 82–124 days old when the lesions were first observed. If we also consider the true positive test results of the GCmcp assay with naïve Lake Sturgeon and White Sturgeon cell lines derived from multiple tissue types (Figure 9), the presence of AciHV-3 sequence data in sturgeon chromosomal sequence datasets (Table 1) as well as our own unpublished results, a more plausible explanation is vertical transmission of an endogenous virus. We hypothesize that AciHV-3 has established a persistent latent infection by integration into the chromosome of sturgeon cells. Furthermore, we posit that the virus has endogenized into the chromosome of germline cells since the virus was found in more than one type of somatic cell (i.e., skin, spleen, gonad). Although there are a number of endogenous herpesviruses within the Herpesvirales order (e.g., Philippine *Tarsier syrichta* herpesviridae-like virus (TsyrHVL) [62]; Teratorn lineage of viruses [63,64]; Marek’s disease virus [65,66,67]), this type of heritable latent infection has only been reported for human herpesviruses 6A and 6B (HHV6) [68,69]. If the hypothesis holds true, then the AciHV-3 virus detected in the juvenile GRH sturgeon could be inherited as a host allele from one or both wild parents and then reactivated causing dermal lesions in the progeny. We present more evidence to support this hypothesis later in the discussion (and in a subsequent paper). As with HHV6, this type of latent infection may mean that virus DNA is detected in asymptomatic, apparently healthy sturgeon when using molecular assays to diagnose AciHV-3. Targeted surveillance of populations using an AciHV-3-specific quantitative PCR assay will provide additional insight into the persistence and prevalence of this virus in wild Lake Sturgeon.

Lesions in GRH-reared Lake Sturgeon were first observed in the fall of 2017. They had not been observed in Lake Sturgeon stocks reared annually at the hatchery prior to 2017 and have not been seen since their last occurrence in the 2018 year class. Recrudescence of latent herpesvirus infections is typically associated with changes to host biology including smoltification, spawning or gonad differentiation and/or fluctuations in environmental conditions such as water temperature, salinity or pH [24,25,61]. Lesion prevalence was higher in the GRH 2018 year class populations (2017YC, 3–19% vs. 2018YC, 31–40% (Figure 3)), which experienced a 6 to 9 °C decrease in water temperature prior to the initial observation of the lesions (Figure S1 in Supplement S1). Although water temperature was stable prior to lesions developing in the 2017YC populations, the sturgeon were experiencing a decrease in water temperature at the time lesion prevalence was determined (Figure S1 in Supplement S1). The increase in lesion prevalence observed in the lower Nelson River isolated sub-group also corresponded to when the hatchery water temperature was reduced to acclimate the yearlings prior to their release in the spring of 2019. The association we observed between the appearance of skin growths and the lower water temperatures in the fall has been reported for other fish alloherpesviruses, including Esocid herpesvirus [70,71], European perch herpesvirus [72] as well as members of the *Ictavirus* (e.g., IcHV-1) [73] and *Salmovirus* (e.g., SalHV-1, SalHV-3) [74] genera. In essence, alloherpesviruses seem to have adapted to the variable body temperature of their poikilothermic hosts by adopting a unique temperature-dependent model for latent virus expression [24]. Therefore, it would not be surprising if temperature was an environmental trigger of AciHV-3 (re)expression.

The apparent reactivation of AciHV-3 produced a mild infection that resulted in the production and eventual regression of epithelial lesions. The seasonal appearance of skin lesions caused by latent alloherpesviruses in adult fish is generally considered a benign process [61]. It provides a mechanism for creating a reservoir of latently infected hosts, as illustrated by the European smelt herpesvirus [75]. In this case, virus-induced skin growths develop in fish experiencing the hormonal stress of spawning. Exogenous virus particles in the lesion are passively released into the environment and transmitted to naive sympatric hosts at the spawning site [75]. This virus transmission pathway has also been proposed for AciHV-1 in spawning adult Lake Sturgeon [15,16], which is significant given that AciHV-1 is the closest genetic relative to AciHV-3. Mortality due to these tumor-associated viruses is typically only observed when the virus is expressed in larvae and juveniles, as has been described for CyHV-1 (i.e., carp pox HV) [76], SalHV-2 [77,78], SalHV3 (i.e., Lake Trout epizootic epitheliotropic disease virus) [79,80] and flounder herpesvirus [81]. The absence of mortality, despite the young age of the sturgeon in this study, in conjunction with the temperature and handling stress experienced by these populations underscores the apparent benign nature of an AciHV-3 infection. The association of these viruses with their hosts over long timescales may have led to the domestication and exaptation of AciHV-3 (possibly for host protection), as has been reported for other viruses [82].

Lesion prevalence varied between progeny of parents from the three spawning sites and between families of progeny originating from broodstock from the same river. These results imply that genetic-based differences in Lake Sturgeon resistance to AciHV-3 may exist and could play a role in whether the latent infection remains quiescent or virus expression is activated. Differences in alloherpesvirus susceptibility have been reported for genetic lineages of White Sturgeon [26] and Common Carp *Cyprinus carpio* L. [83,84,85]. Investigation into the basis for the genetic resistance of cyprinid hosts to cyviruses has revealed similarities in immune suppression and evasion mechanisms used by alloherpesviruses and herpesviruses [86,87]. For example, CyHV-3 appears to inhibit the host interferon type 1 response required for a robust anti-viral innate immune response (immune suppression) and encodes a functional homologue of IL-10 that is expressed during latent infection (immune evasion) [83,84,85,88]. As with other herpesviruses, AciHV-3 disease incidence and pathogenesis are likely to be influenced by multiple factors, including, but not limited to, the genetic signature of each host.

AciHV-3 is a novel virus and likely represents a new species of alloherpesviruses. The taxa form a unique evolutionary lineage that descends as a monophyletic clade from an ancestral node shared with AciHV-1 taxa. Collectively, the AciHV-1 and AciHV-3 clades appear to be isomorphic with the taxonomic level of genus within the *Alloherpesviridae* family. Since AciHV-1 is an unclassified alloherpesvirus, this grouping would represent a new genus for this family. The apparent species- and genus-level clusters are strongly supported in the Bayesian inference of phylogeny conducted with the concatenated core protein sequences (Figure 8). We are proposing that the ICTV consider adopting the true species classification of Acivirus acipenseridallo3 and the formal genus designation of *Acivirus*. The more distant genetic relationship between members of the proposed *Acivirus* genus and AciHV-2 is reflected in the topology of the concatenated core protein tree (Figure 8) and lower pairwise mcp sequence identity (23–26%). The former is consistent with a potential host shift of an alloherpesvirus from catfish to sturgeon.

AciHV-3 is present in at least six species of sturgeon. In addition to Lake Sturgeon, we detected the virus in Shortnose Sturgeon, White Sturgeon, Atlantic Sturgeon, Sterlet Sturgeon and Pallid Sturgeon. Notably, the retracted sequences [10] reported by Kelley et al. [17] for AciHV-1, WSHV-99-ID and WSHV-03-CA were 99–100% identical to the corresponding region of the DNA polymerase gene from the AciHV-3 isolates reported here, suggesting that additional isolates of AciHV-3 may be present in White Sturgeon from California and Italy. The high nucleotide and amino acid sequence identity of the full-length mcp among the Sterlet and Lake Sturgeon AciHV-3 taxa (i.e., >99%) suggests an ancient host–pathogen relationship. The >95% identity across the 493 bp region of the mcp gene from all six sturgeon species provides initial support for the idea that the virus is orthologous across all sturgeon species. It would be useful to test samples from members of the *Polyodontidae* family to see if the host range can be expanded beyond the *Acipenseridae* family to the order Acipenseriformes. The results could provide additional insight into the paleovirological profile of the AciHV-3 lineage.

Host-specific adaptation of AciHV-3 is evident in the genotyping tree topology and is likely influenced by the more recent biogeographic isolation of extant sturgeon species. As with the sturgeon mimivirus Namao virus [89], North American lineages of AciHV-3 appear to display biogeographic isomorphism with continental drainages that are naturally separated by drainage divides. More extensive sampling is required, but in this scenario the LS AciHV-3 lineage would only be found in the Hudson Bay drainage basin, whereas the Pallid Sturgeon and White Sturgeon AciHV-3 lineages would be confined to the Mississippi River drainage and Pacific basins, respectively. Within each drainage basin, the host and virus would co-evolve, and this allopatric co-speciation would be mirrored in their respective species phylogenies. Thus, congruence in the topology of host and virus phylogenies could provide support for a history of co-divergence, as described for other herpesviruses [90]. The evolutionary relationships within the *Acipenseridae* family have yet to be resolved, but key observations have consistently emerged from phylogenetic reconstructions—the European and Atlantic Sturgeon clade descends from the most basal node, followed by the Scaphirhynchus clade, which is basal to the remaining species that cluster in Atlantic and Pacific Acipenser clades [4]. This general branching pattern is observed in our AciHV-3 genotyping tree. For example, the Atlantic and Pallid Sturgeon AciHV-3 descend from the two most basal nodes of the tree, whereas AciHV-3 from acipensers of the Atlantic and Pacific clades descend from a strongly supported common ancestor in lineages that are monophyletic by host (Figure 9). Herpesviruses are known for their host specificity, but in this case, more information is required to understand whether or not the apparent host–virus co-diversification has resulted in AciHV-3 specificity for distinct sturgeon species or if inter-species transmission of this virus is possible in the absence of geographic constraints. We expect that the synchronous development of AciHV-3 with their hosts over millions of years and their potential endogenous lifestyle have likely resulted in strict host specificity due to extensive mutual co-adaptation.

The apparent slow rate of AciHV-3 evolution is consistent with an endogenous virus lifestyle in which an ancestral exogenous virus integrated into a germline chromosome. Virus endogenization, once thought to be a rare event, is actually common, with representatives from every known group of viruses [91]. Germline integration is still considered to be a rare event, with only one known representative from the Herpesvirales order—HHV6. Once endogenous viruses are fixed in a population, they evolve at the host rate of evolution and retain similarity to their ancestral exogenous virus for many millions of years [91]. The slow rate of AciHV-3 evolution is evident in the unusually high DNA and protein sequence conservation observed among AciHV-3 isolates from six biogeographically isolated sturgeon species. The low inter-host virus sequence diversity would follow an ancestral integration event if the AciHV-3 lineages expanded via intra-host species reproduction and vertical transmission (rather than exogenous virus replication and horizontal transmission). This type of virus evolution is evident in HHV6 phylogenies as clades with very short intra-clade branch lengths that are separated from each other by long inter-clade branches [92]. Both patterns are evident in the AciHV-3 genotyping tree, providing initial support for an endogenous virus established by germline integration. The question then becomes about when the integration event occurred relative to the timeline of host speciation and/or geographic isolation. Did a single integration event occur in a common ancestor to the Acipenseriformes order or *Acipenseridae* family or does each acipenser species represent an independent integration event? Sampling polyodonts and identifying the chromosome and integration site(s) of the virus in each acipenser species will be critical to addressing these questions. Interpretation of the results will likely be confounded by differences between the host species with respect to ploidy and the number of chromosomes.

Capsid structure and assembly are conserved across the three families of the Herpesvirales, and this is used to support the idea of a common evolutionary origin [90]. Despite having limited primary sequence similarity across families, the most abundant capsid protein (i.e., major capsid protein) displays a domain organization that is conserved across the order [93]. Fine-scale resolution of the architecture of the Herpes Simplex Virus type 1 (HSV-1) major capsid protein VP5 has revealed three domains that are referred to as the upper (ud), middle and floor domains [94,95]. The HSV-1 (1374 amino acids; 149.1 kDa) and Lake Sturgeon AciHV-3 (1295; 144.5 kDa) major capsid proteins are similar in molecular weight and, as observed previously with other alloherpesviruses [93], the proteins do not share discernable amino acid sequence homology. If we assume that the three domains determined for HSV-1 VP5 exist in the LS AciHV-3 mcp, then we can postulate on the biological relevance of the results obtained with the genotyping assay. The AciHV-3 genotyping assay encodes a portion of the mcp that appears to correspond to the central region of the VP5ud (ud is aa 451–1054). This region contains a number of residues responsible for stabilizing the lateral interactions between VP5 subunits assembled in hexon capsomeres that form the faces of the icosahedral capsid [94,95]. Within the 493 bp region of the mcp gene, the genetic diversity among AciHV-3 taxa is clustered into two nucleotide blocks that correspond to the mcp regions aa 629–657 and 761–788 (Table S16 in Supplement S3). Given the unusually high DNA and protein sequence conservation of the AciHV-3 mcp across the six sturgeon species, these residues may be critical for selective adaptation of the virus capsid (e.g., stability) within each host species. Since the function of the capsid is to protect, transport and deliver the virus genome into the host nucleus, such evidence of host–pathogen co-evolution would likely be found within the sequence of the most abundant protein component of the capsid.

Sturgeon aquaculture has increased globally in response to the decline of wild sturgeon stocks, regulated protection of Acipenser species and the ongoing demand for sturgeon products [96,97]. Herpesviruses such as AciHV-3 have the potential to cause lethal disease outbreaks and reduce production success in hatchery-reared populations. Since AciHV-3 homologs likely exist in all sturgeon species, understanding the factors that contribute to disease emergence will be critical to ensuring the sustainability of the sturgeon aquaculture industry. We continue to investigate existing knowledge gaps about this virus to enable the implementation of hatchery management practices that reduce the risk of disease in farm settings. If temperature fluctuations are a contributing factor, as suggested by this study, then the development of mitigation strategies for hatcheries will be an important step—especially in light of the ongoing and predicted future changing climate conditions.

## Figures and Tables

**Table 2:** Pairwise percent identity (similarity) of the full-length major capsid protein amino acid sequence among alloherpesviruses. The maximum and minimum range is provided for viruses within each genera **^1^**.

Virus Taxa/Genera ^1^	Acivirus ^2^		Ictavirus ^3^	Batravirus	Cyvirus
	AciHV-3	AciHV-1			
AciHV-3	99–100 (99.6–100)	-	-	-	-
AciHV-1	31–32 (48)	85–90 (91–94)	-	-	-
Ictavirus	24–25 (42–43)	23–26 (41–45)	51–90 (69–97)	-	-
Batravirus	21–25 (37–40)	20–23 (36–41)	17–22 (35–40)	24–39 (41–58)	-
Cyvirus	18–19 (32–34)	17–18 (31–33)	14–18 (28–34)	30–31 (30–32)	29–76 (47–85)

^1^ See Table 1 for list of taxa, ^2^ Currently unclassified, ^3^ AciHV-2 species are classified under the *Ictavirus* genus.

## Data Availability

The genetic data generated and used in this study have been deposited in the Genbank database of the National Center for Biotechnology Information (see Table 1 for accession numbers). Fish biometric data, lesion prevalence and density data and water temperature profiles are provided in Supplement S1, and genetic data used in the study are provided in Supplements S2 and S3.

## Supplementary Materials

The following supporting information can be downloaded at: https://www.mdpi.com/article/10.3390/pathogens12091115/s1, (1) Supplement S1 (Figure S1: GRH 2017 and 2018 water temperature profile; Tables S1–S4: GRH Lake Sturgeon biometrics when lesions were first discovered in 2017 and 2018; Tables S5 and S6: GRH Lake Sturgeon lesion prevalence in 2017 and 2018; Table S7: intra-fish lesion density in lower Nelson River Lake Sturgeon sub-group); (2) Supplement S2 (Tables S8–S13: DNA and protein sequences of the five alloherpesvirus core proteins from viruses used in the concatenated core protein phylogeny); (3) Supplement S3 (Table S14: alloherpesvirus mcp DNA sequence pairwise identity; Table S15: AciHV-3 DNA and amino acid sequence data for the 493 bp region of the mcp gene; Table S16: Summary of SNP clusters and types of mutations observed in the 493 bp region of the AciHV-3 mcp gene).
